# Rectal and Anal Surgery
*Rectal and Anal Surgery. By Dr. E. Andres, M.D., L.L.D.; and E. W. Andrews, A.M., M.D. 140, pp. Illustrated. (W. T. Keener, Chicago, 1889.)

**Published:** 1891-03-14

**Authors:** 


### THE PRACTITIONER'S BOOKSHELF.

### RECTAL AND ANAL SURGERY.*

The authors of this little book have aimed at (1) showing what are the best modern methods of diagnosis and treatment known to the regular profession; and (2) discovering what are secret methods of the *rectal specialist*, and what their value. The last question, after much research, yields, as might have been anticipated, very little material worth knowing. The first is answered in a clear and simple way; but the limited scope of the work hardly entitles it to rank as a "Manual." The formulary given at the end is a good point, and contains reliable formulæ for the treatment of diseases of the rectum and anus.

*Rectal and Anal Surgery. By Dr. E. Andrews, M.D., L.L.D.; and E. W. Andrews, A.M., M.D. 140, pp. Illustrated. (W. T. Keener, Chicago, 1889.)

